# Transcription Regulation and Genome Rewiring Governing Sensitivity and Resistance to FOXM1 Inhibition in Breast Cancer

**DOI:** 10.3390/cancers13246282

**Published:** 2021-12-14

**Authors:** Yvonne Ziegler, Valeria Sanabria Guillen, Sung Hoon Kim, John A. Katzenellenbogen, Benita S. Katzenellenbogen

**Affiliations:** 1 Department of Molecular and Integrative Physiology, University of Illinois at Urbana-Champaign, Urbana, IL 61801, USA; yziegler@illinois.edu (Y.Z.); valeria.sanabriaguillen@gmail.com (V.S.G.); 2 Department of Chemistry, University of Illinois at Urbana-Champaign, Urbana, IL 61801, USA; kimsh@illinois.edu (S.H.K.); jkatzene@illinois.edu (J.A.K.); 3 Cancer Center at Illinois, University of Illinois at Urbana-Champaign, Urbana, IL 61801, USA; 4 Institute for Genomic Biology, University of Illinois at Urbana-Champaign, Urbana, IL 61801, USA

**Keywords:** breast cancer, gene networks and rewiring, FOXM1, growth inhibition, resistance

## Abstract

**Simple Summary:**

The oncogenic transcription factor FOXM1 is overexpressed in many cancers and associated with poor patient outcomes. Hence, there is much interest in blocking FOXM1 activity in cancer. We used small molecule inhibitors of FOXM1 to understand how they impact gene regulatory networks in suppressing cancer cell survival and the rewiring of gene networks that occurs when breast cancer cells become resistant to these compounds. Resistant cells showed reversal of the expression of many genes in the FOXM1 network controlling cell cycle progression, DNA damage repair, and apoptosis and also enhanced inflammatory signaling and upregulated HER2 and EGFR pathways. Targeting some of these factors so as to reduce the inflammatory and growth factor-dominant state of the resistant cancer cells should offer promising approaches for suppressing cancer progression and improving treatment of breast cancer.

**Abstract:**

Forkhead box M1 (FOXM1), an oncogenic transcription factor associated with aggressiveness and highly expressed in many cancers, is an emerging therapeutic target. Using novel 1,1-diarylethylene-diammonium small molecule FOXM1 inhibitors, we undertook transcriptomic, protein, and functional analyses to identify mechanisms by which these compounds impact breast cancer growth and survival, and the changes that occur in estrogen receptor (ERα)-positive and triple negative breast cancer cells that acquire resistance upon long-term treatment with the inhibitors. In sensitive cells, these compounds regulated FOXM1 gene networks controlling cell cycle progression, DNA damage repair, and apoptosis. Resistant cells showed transcriptional alterations that reversed the expression of many genes in the FOXM1 network and rewiring that enhanced inflammatory signaling and upregulated HER2 or EGFR growth factor pathways. ERα-positive breast cancer cells that developed resistance showed greatly reduced ERα levels and responsiveness to fulvestrant and a 10-fold increased sensitivity to lapatinib, suggesting that targeting rewired processes in the resistant state may provide benefits and prolong anticancer effectiveness. Improved understanding of how FOXM1 inhibitors suppress breast cancer and how cancer cells can defeat their effectiveness and acquire resistance should be helpful in directing further studies to move these agents towards translation into the clinic.

## 1. Introduction

FOXM1 is an oncogenic transcription factor associated with cancer aggressiveness and poor patient survival [1,2]. It is overexpressed in a broad range of cancer types, including breast cancer, ovarian cancer, glioblastoma, pancreatic cancer, prostate cancer, and GI tract cancers [3,4,5,6]. Its presence is associated with increased cancer cell proliferation and metastasis [7,8,9,10,11]. Hence, there should be therapeutic benefit from inhibiting the activity of FOXM1 in these cancers.

We have identified small molecule inhibitors of FOXM1 that suppress the activity of this protein and reduce the level of FOXM1 in breast cancer cells and tumors. These novel 1,1-diarylethylene-diammonium compounds bind directly to the FOXM1 protein and increase its proteasomal degradation. NB73 and NB115 compounds were synthesized after verification of FOXM1 target engagement and structural optimization of hits from a more than 130,000 member chemical library of compounds. These diamine compounds were shown to reduce the level of FOXM1 mRNA and protein and to inhibit the same FOXM1-signature gene expressions and impact the same gene ontologies as did FOXM1 knockdown with siRNA. They inhibited the proliferation of a variety of breast cancer cells expressing FOXM1 and increased apoptosis [11]. Dose ranges from 0.1 to 10 µM were tested in cells in vitro, and IC50 concentrations for suppression of cell proliferation were 0.7–1 µM for NB73 and NB115 [11]. Furthermore, both compounds showed good pharmacokinetics and long half-lives in mice after s.c. administration [11], and they suppressed breast tumor xenograft growth and the expression of tumor FOXM1-regulated genes [11] and tumor metastasis [8]. We believe that both inhibition of FOXM1 and reduction in the level of FOXM1 in the cancer cells by these inhibitors contribute to their suppression of FOXM1 activity. Consequently, they diminish the growth and metastasis of breast cancers in preclinical tumor models [8,11].

Since FOXM1 is an emerging therapeutic target in breast cancer, we have explored, in this study, the gene networks and signaling pathways impacted by these inhibitors in inhibitor-sensitive breast cancer cells, and we have investigated cancer hallmarks and signaling pathways that are altered and rewired when cells maintained in the presence of these compounds for long periods of time develop resistance to these agents.

What is of note is that we observed that breast cancer cells with acquired resistance upregulate interferon signaling pathways and/or TNFα inflammatory signaling, as well as growth factor regulated pathways while downregulating estrogen receptor (ERα), which enable them to overcome the growth inhibition initially brought about by these compounds. Of interest, these same resistance pathways are known to be altered when breast cancers become resistant to other cancer therapeutic agents such as CDK4/6 inhibitors, endocrine agents such as tamoxifen, or DNA damaging radiation or chemotherapies [12,13,14,15,16,17,18], although our findings also reveal that the FOXM1 inhibitors show some distinct differences in mechanisms of resistance. The observations reported here delineate mechanisms by which FOXM1 inhibitors can suppress ER-positive and triple negative breast cancer (TNBC) and the pathways that become altered in resistance. Analysis of clinical data indicates that these changes appear to be of relevance in predicting a poorer clinical outcome in patients with distinct subtypes of breast cancer. The findings also uncover possible approaches for extending efficacy in the treatment of breast cancer.

## 2. Results

### 2.1. Transcriptional Effects of FOXM1 Inhibition in Sensitive and Resistant Breast Cancer Cells

In order to understand the biological mechanisms important in the inhibition of breast cancer cell growth and progression by FOXM1 inhibitors, we studied ER-positive and triple negative breast cancer cells sensitive to FOXM1 inhibitors, and we also developed long-term treated cells that acquired resistance to growth suppression by these inhibitors.

**Figure 1:**
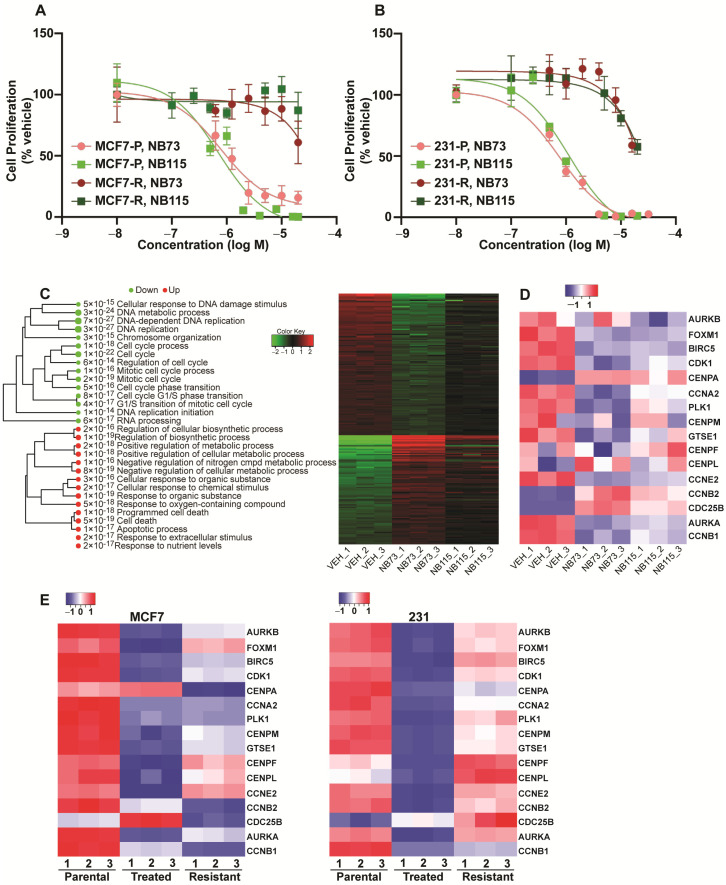
Effects of FOXM1 inhibitors on the proliferation and gene expression profiles of parental MCF7 and MDA-MB-231 cells and of cells grown long-term (>6 months) in the presence of inhibitors that acquired resistance to growth suppression. (**A**,**B**) Proliferation of parental (P) and resistant (R) cells was monitored after treatment for 5 days with the indicated concentrations of compounds NB73 or NB115. Assays were conducted in triplicate. Values are mean ± SEM. (**C**) Heatmap of differential RNA-seq gene expression analysis showing genes that were upregulated or downregulated >2-fold and with FDR < 0.05 in the treated groups (4 µM NB73, 4 µM NB115, MCF7 cells for 9 h) compared to Vehicle (n = 3 samples per group). Enrichment tree denotes GO terms associated with genes that were upregulated (red circles) and downregulated (green circles). Size of circles denotes magnitude of GO term *p* value. (**D**) Expression of FOXM1 target genes in parental vehicle treated MCF7 cells and in MCF7 cells treated with NB73 or NB115 for 9 h. Heat map shows 3 samples per group. (**E**) Regulation of FOXM1 signature genes is lost for most genes in the resistant cells. Expression of FOXM1 signature genes is shown in control MCF7 and 231 parental cells (P), in cells treated with NB73 for 24 h (T), and in resistant cells (R) maintained in 10 µM NB73 (n = 3).

In order to develop breast cancer cells with acquired resistance to our FOXM1 inhibitors, ER-positive (MCF7) and triple negative (MDA-MB-231) breast cancer cells were exposed to increasing concentrations of inhibitor (NB73 or NB115) over a 6 to 9-month period, until we reached a point where cells grew well and maintained good viability in a maintenance dose of 10 µM compound. Pooled resistant cells were utilized, and we used at least three different derived cell lines in each experiment. As observed in Figure 1A,B, IC50 concentrations for inhibition of cell proliferation in parental sensitive cells were 0.8 ± 0.12 µM for NB73 and 0.9 ± 0.15 µM for NB115 (n = 3) in contrast to >80 µM in resistant MCF7 or >30 µM in resistant 231 cells. No statistical difference in the IC50 values for suppression of proliferation of parental MCF7 or 231 cells by NB73 or NB115 treatment was found (Supplementary Table S2). Thus, MCF7 and 231 parental cells respond similarly to these two compounds, with NB73 and NB115 showing similar potencies in the suppression of proliferation. IC50 values for resistant cells could not be determined statistically because suppression of proliferation was incomplete at the highest dose (20 µM) we could examine in the resistant cells because of compound solubility. We conducted Sanger DNA sequencing on all *FOXM1* exons by comparing parental sensitive to resistant cells. This revealed that the transcribed sequences of the *FOXM1* gene were identical in all of these cells, eliminating the possibility that resistance came about through a mutation in the protein encoding regions of the *FOXM1* gene.

Differential RNA-seq gene expression analysis revealed that the inhibitors NB73 and NB115 exhibited similar patterns of upregulation and downregulation of gene expression (Figure 1C) and that the genes downregulated by short-term treatment (green circles) were enriched for GO terms relating to cell cycle function, DNA replication, DNA repair, cell cycle phase transition, and mitotic processes (Figure 1C). In the upregulation group (red circles), we observed GO terms denoting apoptosis or cell death, response to nutrient levels, and cellular response to chemical stimuli. A heat map showing the expression of 16 known FOXM1 target genes, including FOXM1 itself (Figure 1D), indicates downregulation of most target genes by NB73 and NB115 except for three genes (CENPA, CDC25B, and CCNB2) that were upregulated by both of these compounds. Changes in mRNA levels of important growth associated genes were confirmed by RT-qPCR. As observed in Supplementary Figure S1, NB73 and NB115 exposure for 24 h greatly downregulated expression of genes associated with cell cycle progression (e.g., CCNE1, RB1, CDC25A, E2F1, CDKN2C, MCM2, CDK2, RRM2, and PCNA), including the expression of FOXM1 itself, while upregulating the expression of CDKN1A, a gene associated with suppression of proliferation. Figure 1E shows that FOXM1 target gene downregulations seen in short-term inhibitor treated MCF7 or 231 cells are lost for almost all genes in the resistant cells (see Supplementary Tables S3 and S4 for statistical analyses of Figure 1D,E data).

### 2.2. Comparison of Gene Regulations in Cells Sensitive or Resistant to FOXM1 Inhibitors

**Figure 2:**
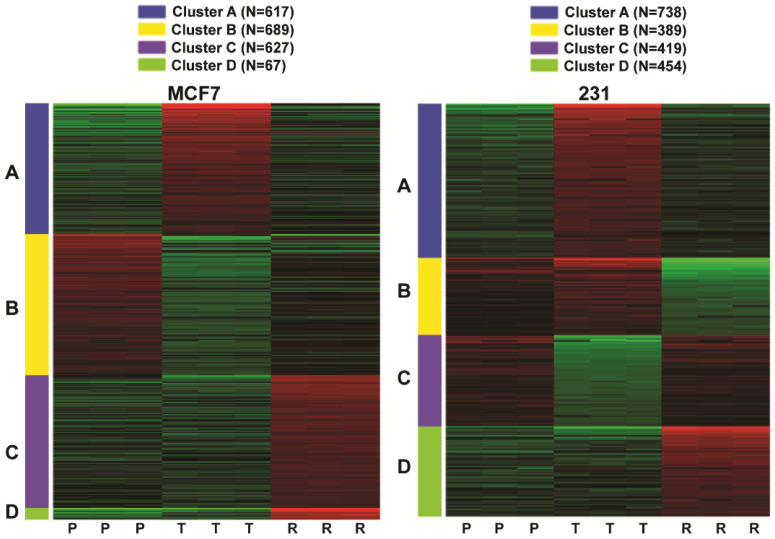
Three-way comparison of RNA-Seq gene expression analysis in parental (P), NB73 short-term treated (T), or NB73 resistant (R) cells. MCF7^P^ or 231^P^ cells were short-term treated with 4 µM NB73 (T) or Vehicle (P) for 24 h; MCF7^R^ or 231^R^ cells were continued in their maintenance dose of 10µM NB73. Heatmaps show genes with >2-fold expression changes and FDR < 0.05 in differential gene expression analysis. iDEP and K-means clustering analysis revealed four distinct clusters and patterns (A–D) of gene regulations in parental cells (P) versus treated (T) versus cells resistant (R) to NB73. Three parental, three treated, and three resistant samples were analyzed for each cell type.

iDEP software [19] and K-means clustering within the iDEP software enabled us to compare and classify gene regulations in parental (P) control–vehicle treated cells, short-term treated (T) inhibitor sensitive cells, and inhibitor resistant (R) MCF7 or 231 cells into four distinct groupings or clusters, A–D (Figure 2 and Table 1), based on the pattern of up or downregulation observed for genes in the three cell conditions (P, T, and R). Cluster A contains genes (N = 617 for MCF7 and N = 738 for 231 cells) upregulated in parental cells short-term treated with NB73 (T vs. P) that showed decreased expression and returned to parental vehicle expression levels in resistant cells (R vs. P). For both MCF7 and 231 cells, these included genes associated with TNFα signaling via NFκB, MTORC1 signaling, hypoxia, the unfolded protein response, the p53 pathway, and apoptosis. Further examination of some of these Hallmark Pathways shown as Venn diagrams in Supplementary Figure S2 indicates substantial overlap in gene expression regulations in these hallmarks in MCF7 and 231 cells in response to NB73. Cluster B genes in 231 cells (n = 389) were similar in Hallmark Pathways to those in Cluster A and also showed some upregulation in treated cells (231^T^ vs. 231^P^ cells), but they were more highly expressed in parental cells and were markedly downregulated in resistant cells (231^R^ vs. 231^P^ or 231^T^ cells). These included Hallmark Pathways TNFα signaling via NFκB, epithelial mesenchymal transition (EMT), and inflammatory response. Cluster B genes (N = 689 for MCF7) and cluster C genes (N = 419 for 231 cells) were downregulated by short-term (24 h) NB73 treatment and showed partial (MCF7) or full reversal (231) in resistant cells. In both cells, these genes (see Table 1) were associated with the cell cycle, E2F targets, G2/M checkpoint, MTORC1 signaling, Myc targets, and DNA repair, genes that are well known to be under FOXM1 control.

What is of note is that cluster C and D genes in MCF7 cells (N = 627 and 67) and cluster D genes in 231 cells (N = 454) were upregulated only in the resistant cells. These rewired genes (Figure 2 and Table 1) included genes associated with interferon alpha and gamma response, epithelial mesenchymal transition (EMT), the p53 pathway, and TNFα signaling via NFκB. Of note, the upregulation of interferon alpha and gamma signaling we observed in ER-positive NB73-resistant cells is also reported to be observed in endocrine-resistant ER-positive breast cancers [12].

### 2.3. Increased Interferon Inflammatory Gene Expressions and Signaling in ER-Positive Cells with Inhibitor Resistance

**Figure 3:**
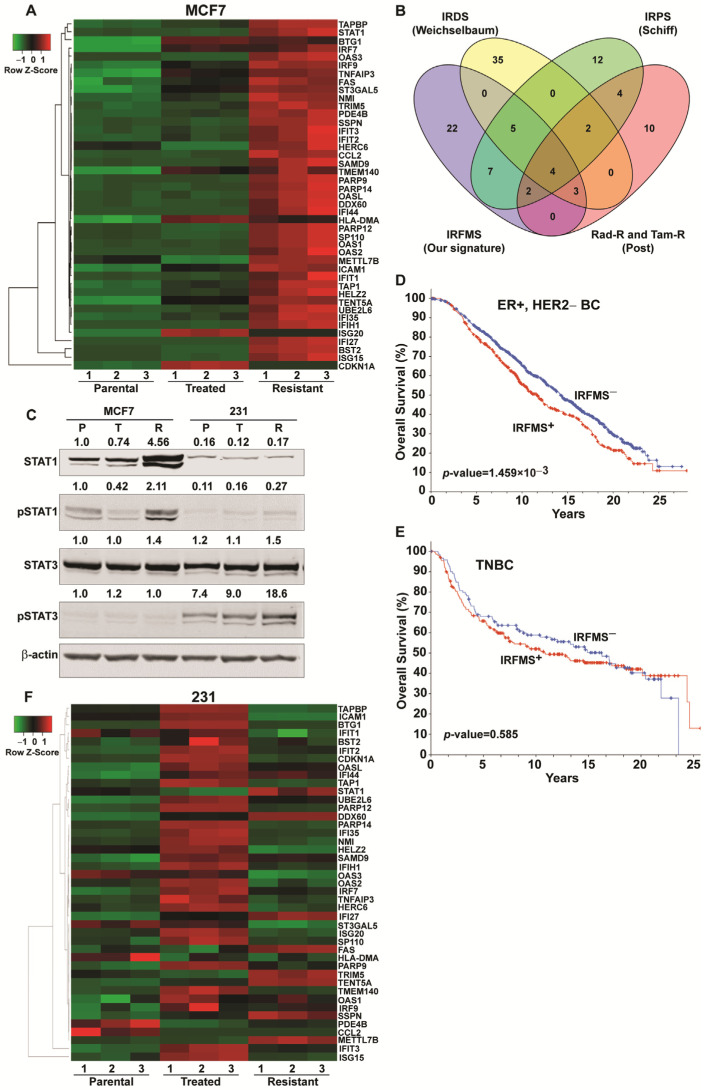
Altered interferon and inflammatory gene expression signaling in FOXM1 inhibitor resistant cells. (**A**) Expression of our IRFMS 43 signature genes in parental (P), treated (T), and resistant (R) MCF7 cells. (**B**) Venn diagram showing genes in our Interferon-Related FOXM1 Inhibitor Resistance Signature (IRFMS) and their overlap with interferon-related signatures reported for CDK4/6 inhibitor Palbociclib resistance (IRPS, Schiff) [12], tamoxifen and radiation resistance (Post) [17], and DNA damage repair/chemotherapy and radiation resistance (IRDS, Weichselbaum) [18]. (**C**) Western blots showing levels of STAT1, pSTAT1, STAT3, and pSTAT3 in MCF7 or 231 parental (P), 24 h NB73 treated (T), and NB73 resistant (R) cells. Numbers above the blot indicate level corrected for loading control β-actin. Level in MCF7^P^ cells is set at 1.0. Original blots see Figure S3. (**D**) IRFMS predicts poor prognosis in patients with ER-positive, HER2-negative breast cancer. Kaplan–Meier curves of overall survival of patients with ER-positive, and HER2-negative breast cancer in the METABRIC dataset (n = 1236) stratified by the mean IRFMS score as described in Methods. Logrank *p*-value is shown. (**E**) Interferon Related FOXM1 Inhibitor Resistance Signature (IRFMS) does not predict prognosis in patients with triple negative breast cancer. Kaplan–Meier curves of overall survival are shown, n = 320 from METABRIC. Clinical samples are stratified by the mean IRFMS score as described in Methods. Logrank *p*-value is shown. (**F**) Expression of our IRFMS 43 signature genes in MDA-MB-231 parental (P), 24 h short-term NB73 treated (T), and resistant (R) cells.

An exciting observation in the ER-positive breast cancer cells was the marked alteration in interferon (IFN) inflammatory-related hallmark gene expressions and increases in associated proteins such as STAT1 and active pSTAT1 that accompany the change from sensitivity to resistance to FOXM1 inhibitors (Figure 3). Based on our observations, we derived an Interferon-Related FOXM1 inhibitor resistance Signature (IRFMS) of 43 genes, as detailed in Materials and Methods Section 4.7. More than 80% of the 224 genes in the Molecular signatures Database MsigDB v7.4 Hallmark gene set for interferon alpha and gamma response were represented in our MCF7 RNA-Seq data, and of these, 43 genes were upregulated more than 2-fold with *p* < 0.05. These 43 genes were defined as our IRFMS, and their names and expression in parental, treated, and resistant cells are shown in Figure 3A. Notably, our IRFMS shows considerable, but not complete, overlap with the genes observed in ER+ breast cancer cells and tumors that have become resistant to other therapies reported by others (Figure 3B). FOXM1 inhibitor resistance showed a 18/36 gene overlap with Schiff palbociclib CDK4/6 inhibitor resistance signature, IRPS [12], a 12/49 gene overlap with the Weichselbaum DNA damage resistance signature, denoted IRDS [18], and a 9/25 gene overlap with post-radiation and tamoxifen resistance signature [17]. The 18 genes in our IRFMS that overlap with the IRPS-Schiff signature are IFI44, IFI27, IFIT1, IFIT3, OASL, IFI35, STAT1, HERC6, IRF7, SAMD9, SP110, IFIH1, PARP9, ISG15, PARP12, IRF9, OAS2, and DDX60. The 12 genes in our IRFMS that overlap with the Weichselbaum IRDS are IFI44, IFI27, IFIT1, IFIT3, OASL, IFI35, STAT1, HERC6, IRF7, OAS1, OAS3, and BST2. The nine genes in our IRFMS that overlap with the Rad-R and Tam-R-Post signature are IFI44, IFI27, IFIT1, IFIT3, OAS1, OAS3, BST2, OAS2, and DDX60. Of interest, IFI44, IFI27, IFIT1, and IFIT3, four members of the interferon inducible gene family, are present in all four interferon response gene signatures with resistance to different agents—FOXM1 inhibitor, CDK4/6 inhibitor palbociclib, DNA damage, and tamoxifen and radiation resistance. These IFI and IFIT genes appear to play important and often distinct roles in various aspects of immune function and drug resistance in cancer [20]. The overlaps in our IRFMS signature with genes in the different resistance signatures and the 22 genes present only in our IRFMS are listed in Supplementary Table S5.

While STAT1 RNA (Figure 3A) and protein (Figure 3C) levels were reduced in MCF7 cells with short-term FOXM1 inhibitor treatment (MCF7^T^ vs. MCF7^P^ cells), STAT1 RNA (Figure 3A) and STAT1 and pSTAT1 protein levels were markedly elevated above parental cells in the resistant cells (Figure 3C). By contrast, STAT1 protein levels were much lower in 231 cells than in MCF7 cells, and they changed little with short-term NB73 treatment or with resistance (Figure 3C). Notably, however, pSTAT3 levels were higher in 231 cells than in MCF7 cells, and pSTAT3 was elevated 2.5-fold in 231 resistant cells (Figure 3C). Upregulated and phosphorylated STAT1 and STAT3 are known to promote breast cancer progression and drug resistance [21] and high levels are associated with poor outcomes and shorter survival in a variety of cancers [22].

### 2.4. The IRFMS Predicts Poor Clinical Outcome in Patients with ER-Positive Breast Cancers

In order to extend our findings in breast cancer cells to primary human breast cancer, we used Kaplan–Meier analysis to examine the relationship between breast cancer patient survival and our IRFMS gene signature. Our analysis included 1236 ER-positive, HER2-negative (ER^+^, HER2^−^) primary breast cancer samples in cBioportal from the METABRIC database with patients followed over 25 years. As observed in Figure 3D, expression of our IRFMS gene signature was associated with a significantly less good overall survival for patients with ER-positive, HER2-negative breast cancer (Logrank *p* = 1.459 × 10^−3^). As expected and in contrast to the highly significant association between the IRFMS signature and patient outcome with ER-positive breast cancers, we did not observe a significant association (Logrank *p* = 0.585) between our IRFMS signature and overall survival in patients with TNBC. Likewise, most of the IRFMS signature genes that showed markedly upregulated expression in MCF7 resistant cells (Figure 3A) did not show elevated expression in resistant triple negative 231 cells compared to parental 231 cells (Figure 3F).

### 2.5. Downregulation of ESR1 and Upregulation of HER2 and EGFR Pathways in Resistance

**Figure 4:**
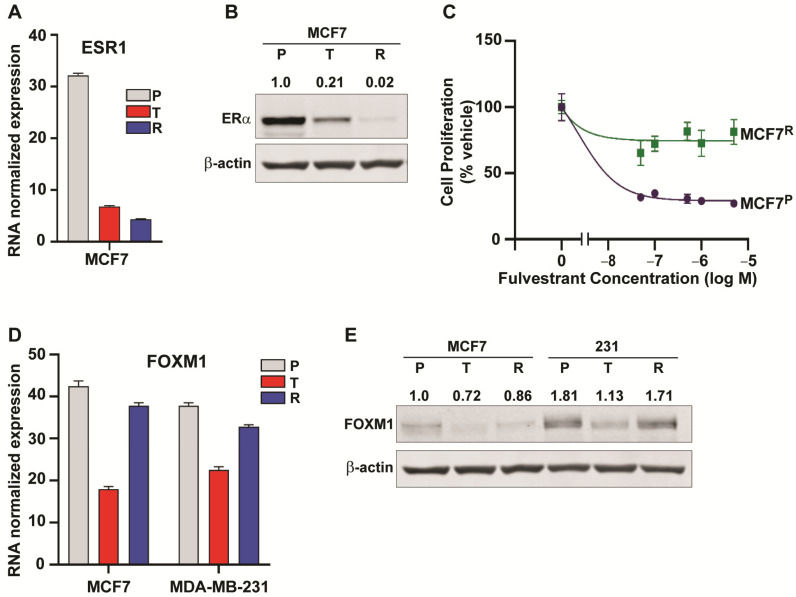
ESR1 and FOXM1 in parental, 24 h inhibitor treated, or resistant breast cancer cells. (**A**) ESR1 RNA expression in MCF7 P, T, and R cells. (**B**) Analysis of ERα protein by Western blot in MCF7 P, T, and R cells. Numbers above the blot indicate level corrected for loading control β-actin. Level in MCF7^P^ cells is set at 1.0. (**C**) Sensitivity to Fulvestrant in parental MCF7^P^ cells and in FOXM1 inhibitor resistant MCF7^R^ cells. Dose–response curves for inhibition of cell proliferation by Fulvestrant are shown. (**D**) FOXM1 RNA expression in MCF7 and in MDA-MB-231 P, T, and R cells. (**E**) Analysis of FOXM1 protein level by Western blot. Numbers above the blot indicate level corrected for the loading control β-actin. Original blots see Figure S3. Level in MCF7^P^ cells is set at 1.0.

Short-term (24 h) treatment with NB73 resulted in a decrease in ESR1 RNA and protein in MCF7 cells (Figure 4A,B). The ESR1 level was further greatly diminished to less than 5% of the parental in MCF7 resistant cells and, as a consequence, the MCF7^R^ cells became almost completely insensitive to growth suppression by the antiestrogen Fulvestrant (Figure 4C). By contrast to what was observed for ESR1, FOXM1 levels in both MCF7 and 231 cells decreased upon short-term (24 h) treatment with NB73, but levels of FOXM1 RNA and protein in both types of resistant cells returned to close to that of parental cells (Figure 4D,E). A similar pattern for FOXM1 was also observed in another TNBC, MDA-MB-436 cells, with acquired resistance to the inhibitor.

**Figure 5:**
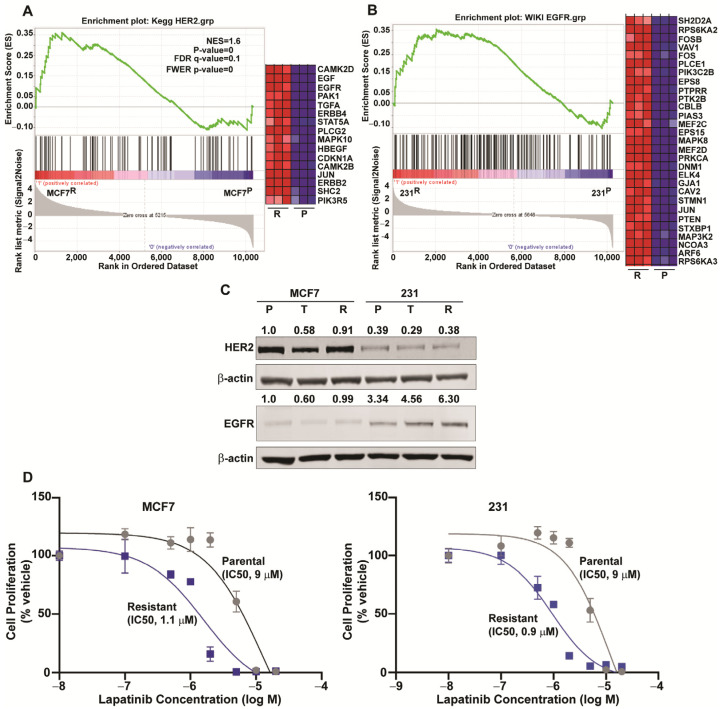
Alterations in growth factors and their pathway gene expressions in FOXM1 inhibitor sensitive and resistant breast cancer cells, and the enhanced sensitivity of resistant cells to lapatinib. (**A**,**B**) GSEA enrichment plots for HER2 and EGFR pathway genes showing elevated expression of these genes in resistant versus parental cells. A, MCF7 cells; B, 231 cells. (**C**) Western blot analysis of HER2 and EGFR protein levels in MCF7 and 231 parental (P), short-term (24 h), treated (T), and resistant (R) cells. Numbers above the blots show level corrected for the loading control β-actin. Level in MCF7^P^ cells is set at 1.0. Original blots see Figure S3. (**D**) Dose–response cell proliferation assays showing increased sensitivity of FOXM1 inhibitor resistant cells to lapatinib. MCF7^P^ and MCF7^R^ cells (left) and 231^P^ and 231^R^ cells (right) were treated with control vehicle or the indicated concentrations of lapatinib for 5 days. Values are mean ± SEM of triplicate assays.

As observed in Figure 5A,B, FOXM1 inhibitor resistant MCF7 and 231 cells showed enrichment for genes associated with HER2 or EGFR signaling, respectively. EGFR protein was higher in parental 231 compared to MCF7 cells, and EGFR was approximately doubled in 231^R^ versus 231^P^ cells (Figure 5C). Moreover, HER2 protein was reduced from the parental level by short-term (24 h) treatment with NB73, whereas HER2 was essentially restored back to the parental level in resistant MCF7 and 231 cells. Associated with the elevated expression of EGF, EGFR, and HER2 in resistant cells, the resistant MCF7 and 231 cells showed an approximately 10-fold increase in sensitivity to lapatinib for suppression of proliferation compared with their parental cells (Figure 5D). These findings are consistent with an increased involvement of these growth factors and their pathway regulations when cells become resistant to FOXM1 inhibitors.

### 2.6. Changes in Genes Associated with the Cell Cycle and Energy Generation in Inhibitor Sensitive and Resistant Breast Cancer Cell States

**Figure 6:**
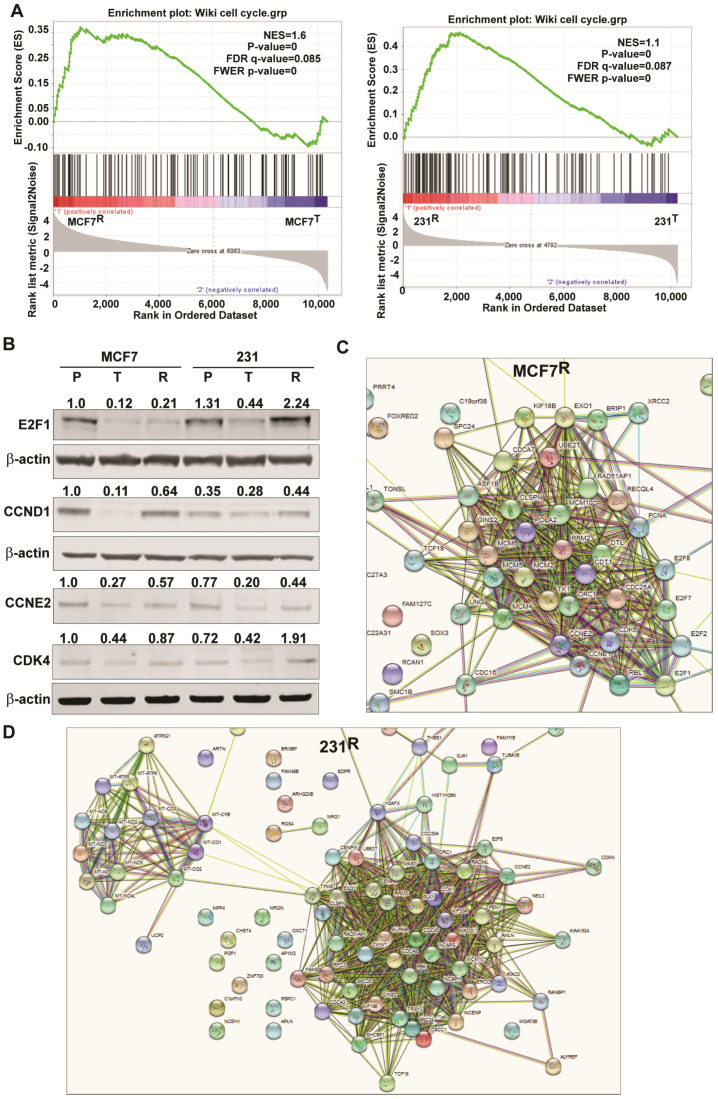
Enrichment of cell cycle genes and their protein expression in resistant MCF7 and 231 cells compared to short-term treated cells. (**A**) GSEA enrichment plots for cell cycle group genes in MCF7^R^ versus MCF7^T^ (left) and 231^R^ versus 231^T^ (right) cells. (**B**) Western blots showing relative levels of the indicated proteins in parental (P), 24 h treated (T), and resistant (R) cells. Numbers above the blots show level corrected for the loading control β-actin. Level in MCF7^P^ cells is set at 1.0.Original blots see Figure S3. (**C**,**D**) iDEP and STRING analyses delineating gene interaction hubs in MCF7 and 231 NB73 resistant cells versus short-term (24 h) NB73 treated cells.

**Figure 7:**
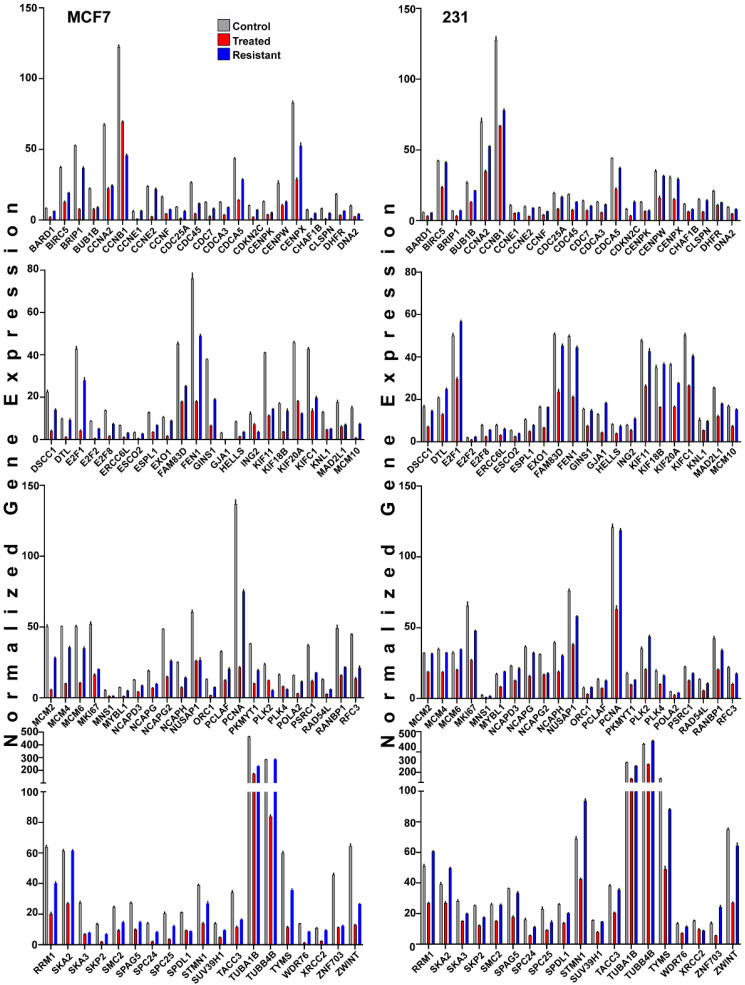
Comparison of the expression of 85 cell cycle related genes in MCF7 and 231 cells with FOXM1 inhibitor sensitivity or resistance. Gene expression in control parental (grey), short-term 24 h treated (red), and resistant (blue) cells is shown. Genes are arranged alphabetically and represent those in the iDEP cell cycle gene category. Each bar is the mean from three separate assays. Error bars were less than 10% of the mean and are not shown for clarity. Trends overall show reduced gene expression in short-term (24 h) treated cells (red) and their reversal with increased expression back toward control parental (grey) level of expression in NB73 resistant (blue) cells. Gene-wise statistical analysis for each of the 85 cell cycle related genes in MCF7 and in 231 parental, short-term treated, and resistant cells is presented in Supplementary Table S6.

The expression of many genes promoting cell cycle progression was reduced in short-term FOXM1 inhibitor-treated (MCF7^T^ or 231^T^) cells but little or not at all in inhibitor-resistant MCF7^R^ or 231^R^ cells (Figure 6A and Figure 7). In fact, some cell cycle proteins such as E2F1 or CDK4 were 2-fold higher in 231^R^ cells (Figure 6B). Of interest, as shown in the STRING diagrams in Figure 6C,D, both resistant cell lines not only showed elevated expression of well-known cell cycle genes (ex. CCND1, CCNE2, and CDK4) compared to short-term treated cells, but the resistant 231^R^cells also showed elevated expression of a second hub of mitochondrial genes important in regulating oxidative phosphorylation and ATP generation. These mitochondrial genes included MT-CO1, MT-CO2, MT-CO3, MT-ATP6 and 8, and MT-ND2,3,4,5, and 6 (Figure 6D, left), which are critical for energy production and cancer cell survival and likely important for thwarting of therapy effectiveness observed in these FOXM1 inhibitor-resistant cells. Examination of a panel of 85 cell cycle associated genes, as defined in our iDEP analysis and expressed in both MCF7 and 231 cells, revealed their highly downregulated expression by short-term inhibitor treatment and their partial or full restoration of parental expression levels in resistant cells (Figure 7).

In order to evaluate this further, we also used DESeq2 (Bioconductor) analyses to identify Differential Expressed Genes (DEGs) and enriched pathways in DEGs for the comparisons of upregulated gene pathways in MCF7 and 231 cells with short-term (24 h) inhibitor treatment of parental cells and in inhibitor resistant cells. These statistical analyses showed the following for MCF7 and 231 cells. There was higher expression of cell cycle pathway genes in MCF7 Veh versus 24 h inhibitor treated cells (5.1 × 10^−57^ *p* value, n = 163 genes) and for MCF7 inhibitor resistant versus short-term inhibitor treated cells (3.2 × 10^−9^ *p* value, n = 53 genes). For 231 cells, upregulated cell cycle category genes were 2.6 × 10^−23^ *p* value, n = 47 genes for Veh versus 24 h inhibitor treated cells and 3.1 × 10^−7^ *p* value, and n = 38 genes upregulated for inhibitor resistant versus short-term inhibitor rreated cells. We also conducted gene-wise statistical analysis for each of the 85 cell cycle related genes. This is presented in Supplementary Table S6. These analyses confirm the statistically significant changes in cell cycle gene expressions that occur in parental FOXM1 inhibitor sensitive cells and differences in FOXM1 inhibitor resistant cells where cell cycle gene expressions are not downregulated as observed in parental short-term treated cells but rather return to the level of expression more similar to that of control parental cells. Full-uncut Western blots for Figure 3C, Figure 4B,E, Figure 5C and Figure 6B are shown in Supplementary Figure S3.

## 3. Discussion

In this study, we have delineated some of the mechanisms by which FOXM1 inhibitors suppress breast cancer cells and illuminated methods by which the cancer cells change their gene regulations and signaling when they progress from sensitive to acquired resistance upon prolonged exposure to these inhibitors.

### 3.1. Patterns of Gene and Pathway Alterations from FOXM1 Inhibition and the Development of Resistance

Our analyses indicate that initial treatment of breast cancer cells with these inhibitors strongly affects processes related to G1/S and G2/M transitions and S-phase functions such as DNA replication and DNA damage repair, as well as the progression of mitosis. Other studies have shown that FOXM1-deficient cells exhibit delayed S-phase entry, reflecting the importance of FOXM1 at the G1/S transition [23]. FOXM1 also plays a role in almost every part of the DNA damage repair process, and FOXM1-deficient cells accumulate high levels of damaged DNA [24]. The role of FOXM1 in the G2/M transition and in progression of mitosis is well studied, and FOXM1-deficient cells ultimately accumulate spindle defects and undergo mitotic delay and cell death through mitotic catastrophe [25,26]. Thus, our RNA-Seq data and previously reported cell cycle analysis reflecting arrest at G2/M [11] in short-term inhibitor treated cells suggest that the compounds are targeting crucial FOXM1 processes throughout the cell cycle, resulting in accumulation of cells at G2/M, mitotic failure, and marked apoptosis including the unfolded protein response [27].

Transcriptomic alterations and changes in the levels of key proteins were observed to accompany the acquisition of resistance to long-term inhibitor treatment. Many of the genes and gene ontology terms and pathways that were strongly downregulated in the parental cells after short-term treatment with inhibitor were upregulated and restored in resistant cells. However, resistant cells not only restored the previously suppressed expression of many FOXM1 target genes involved in the cell cycle, as well as the expression of FOXM1 itself, but they also showed substantial rewiring of the genome and its transcriptional control. This rewiring accompanied altered inflammatory cell signaling and the greatly upregulated growth factor (HER2 or EGFR) pathway signaling and very diminished ERα presence in ER-positive resistant cells. Resistance to palbociclib and other CDK4/6 inhibitors is also reported to be accompanied by a greatly reduced level of ERα protein in ER-positive breast cancer cells and tumors [12,28].

### 3.2. Common and Contrasting Cellular Alterations Associated with Resistance to FOXM1 Inhibition

Although there has been considerable progress in the development of better and more targeted drugs for breast cancer treatment, the acquisition of resistance to drug treatments is common and limits their long-term use in patients with all subtypes of breast cancer [13,14,15,16,29,30,31,32,33,34]. Our findings in inhibitor sensitive and resistant cells provide new understanding of transcriptional and proteomic perturbations that accompany the acquisition of resistance, and they highlight changes that might provide new therapeutic modalities to target.

An interesting finding was that while the inhibitors had very similar effects on gene expression patterns in short-term treated ER-positive and TNBC cells, the changes that accompanied resistance in the two cell types were quite different. Of note, interferon signaling and the expression of inflammation regulating genes were upregulated in MCF7 cells that had become resistant to the growth suppressive effects of FOXM1 inhibitor, with genes in both interferon γ and interferon α pathways now being overexpressed in MCF7^R^ cells. Notably, several interferon inducible genes in our IRFMS signature were also upregulated in other resistance signatures when resistance to palbociclib, tamoxifen, or radiation developed in ER-positive breast cancer [12,17,18], and these IFI and IFIT genes appear to have important roles in immune function and in tumor progression and drug resistance in cancer [20]. Marked increases in the levels of pSTAT1 and pSTAT3 in the inhibitor resistant MCF7 and 231 cells, respectively, implicate the likely involvement of these STAT family member transcription factors in enhanced and also different inflammatory gene expressions observed in the two resistant cell types. Several earlier publications have noted the involvement of FOXM1 in inflammation in keratinocytes and in other types of cancer [35], and resistance to endocrine therapies in breast cancer has been shown to result in the upregulation of interferon signaling [12]. Of interest, we found that expression of our IRFMS signature is associated with less good survival of patients with ER-positive breast tumors, but it was not prognostic in TNBC patients, consistent with our RNA-Seq findings in resistant TNBC 231 cells where the interferon response was not a highly altered Hallmark Pathway.

Elevated interferon signaling and STAT1 and active pSTAT1 and HER2 expression in ER-positive FOXM1 inhibitor-resistant breast cancer would likely provide an immune suppressive environment that would allow the cancer to progress. The significant association of our IRFMS signature with a much poorer survival in patients with ER-positive primary breast tumors suggests the clinical relevance of our findings derived from breast cancer cell studies. Likewise, increased pSTAT3 and EGFR in resistant TNBC cells would favor growth factor stimulated cancer growth. In this context, it is noteworthy that both ER-positive and triple negative resistant cells showed an approximately 10-fold increased sensitivity to lapatinib for suppression of cell proliferation, suggesting increased involvement of these growth factors and their pathway regulations when cells become resistant to the FOXM1 inhibitors. They also imply that inhibition of growth factor signaling might be useful in overcoming FOXM1 inhibitor resistance.

Our study builds upon existing evidence and suggests that escape from first-line growth inhibitors may commonly occur by making changes in the levels and utilization of cell cycle components, as well as increased involvement of inflammatory signaling, and a switch to HER2 signaling upon acquired drug resistance in ER-positive breast cancer [12,17,18,36]. Indeed, critical and breast cancer subtype-specific relationships are known to exist between proliferation and inflammation in cancer, often involving NFκB that controls the balance between a more aggressive metastasis-promoting state and a more quiescent and controlled cancer state [37]. Targeting some of these factors so as to reduce the inflammatory state of the cancer should offer promising approaches for suppressing cancer progression and improving treatment of primary and recurring breast cancers. Our understanding of how FOXM1 inhibitors suppress breast cancer growth and aggressiveness and how breast cancer cells can defeat their effectiveness and acquire resistance should be helpful in directing further studies to move these agents towards translation into the clinic.

## 4. Material and Methods

### 4.1. Materials, Cell Culture, and Development of FOXM1 Inhibitor Resistant Cells

All breast cancer cell lines were obtained from the ATCC and were maintained and cultured as described [7,38,39]. Cells resistant to the growth suppressive effects of inhibitors were developed by a selection of surviving cells by continuous exposure to increasing concentrations of NB73 or NB115 compounds over a period of 6 to 9 months, starting at 0.1 µM and increasing until a maintenance concentration of 10 µM was reached. Aliquots of pooled resistant cells were frozen and stored in liquid nitrogen. Aliquots were thawed when cells were in culture for more than 10 passages. The cell aliquots were faithful to the original phenotype based on the experiments described herein. All cells were tested for mycoplasma using MycoSensor PCR Assay Kit from Agilent Technologies (Santa Clara, CA, USA). FOXM1 inhibitors were synthesized as described previously [11]. Fulvestrant and lapatinib were obtained from Sigma Aldrich (Burlington, MA, USA). Antibodies against ERα, FOXM1, E2F1, CCND1, CCNE2, CDK4, STAT1, pSTAT1, STAT3, and pSTAT3 were from Cell Signaling Technology (Danvers, MA, USA); the antibody to HER2 was from Abcam; the antibody to EGFR was from Santa Cruz Biotechnology (Dallas, TX, USA); and the antibody to β-actin was from Sigma Aldrich. Further antibody information is provided in Supplementary Table S1.

### 4.2. Cell Viability Assay

WST-1 assay (Roche, Basel, Switzerland) was used to quantify cell viability as described [11]. Absorbance was measured at 450 nm using a VICTOR X5 PerkinElmer 2030 Multilabel Plate Reader. All assays were performed in triplicate and statistically analyzed using Graph Pad Prism 9.0, nonlinear regression, and least squares fit.

### 4.3. Western Blot Analyses

For Western blot analysis, whole-cell extracts were prepared using 1X RIPA lysis buffer (Thermo Fisher, Waltham, MA, USA) supplemented with 1X protease and phosphatase inhibitor cocktail (Millipore Sigma, Burlington, MA, USA). Proteins were separated on 4–12% SDS-PAGE gels and transferred to nitrocellulose membranes. All antibodies were used at a 1:1000 dilution except for β-actin, which used a 1:40,000 dilution with β-actin as an internal loading control in Western blots (see Supplementary Table S1 for detailed information on antibodies). Both IRDye 800 CW goat anti-rabbit secondary antibody (LI-COR, Cat# 926-32211) and IRDye 680 CW goat anti-mouse secondary antibody (LI-COR, Cat# 926-68070) were diluted (1:5000) for incubation with the blots. Band intensities were analyzed with Licor Odyssey Image Studio 5.2 software that avoids saturation, eliminates comparison of multiple exposures, and allows digital analysis of bands of all intensities, with very accurate protein quantification over a broad linear range. All blots shown together were derived from the same experiment and were processed in parallel. Full uncropped images of blots are shown as Supplementary Figure S3. Molecular weight markers were Chameleon Duo markers from Licor (8–260 kDa) or Precision Plus Dual Color Markers from Biorad (37–250 kDa).

### 4.4. RNA-Seq Transcriptional Profiling and Gene Ontology and Pathway Signature and Network Analyses

For gene expression analysis, total RNA was extracted from cells using Trizol reagent and further cleaned using the Turbo DNase and RNAqueous kits (ThermoFisher, Waltham, MA, USA). Cells were treated with Veh (0.1% DMSO) or with the compounds for the times indicated. Once the sample quality and replicate reproducibility were verified, samples from each group were subjected to sequencing. RNA at a concentration of 37.5 ng/µL in nuclease-free water was used for library construction. cDNA libraries were prepared with the mRNA-TruSeq Kit (Illumina, Inc., San Diego, CA, USA). In brief, the poly-A containing mRNA was purified from total RNA, the RNA was fragmented, double-stranded cDNA was generated from fragmented RNA, and adapters were ligated to the ends.

The single-end read data from the HiSeq 4000 were processed and analyzed by using a series of steps. Base calling and de-multiplexing of samples within each lane were conducted with Casava 1.8.2. Reads were trimmed of adapters and low expression data using Trimmomatic version 0.38 [40]. The STAR alignment tool version 2.5.3a was used to align the sequenced reads to the GRCh37 human genome from Ensembl [41]. Gene counts were calculated using subread version 1.5.2 [42]. The edgeR Bioconductor package in R was used for normalization and differential expression analysis. Default normalization methods were used, and trimmed mean of M values or TMM was specifically used to calculate the normalized expression values. This method calculates the weighted trimmed mean of the log expression ratios in a gene-wise fashion [43,44]. We considered genes with fold-change > 2 and *p*-value < 0.05 as statistically significant and differentially expressed.

Heatmapping, hierarchical clustering, differential gene expression analysis, and Gene Ontology (GO) analysis were conducted by using iDEP (integrated Differential Expression and Pathway analysis), a web tool for analyzing RNA-seq data that integrates R and Bioconductor packages [19]. Packages include DESeq2, ggplot2, and limma for identifying differentially expressed genes (DEGs), followed by enrichment analysis using GO. Heatmaps were plotted and hierarchical clustering was performed using iDEP or Heatmapper [45]. Overrepresented gene ontology (GO) biological processes were determined by the web-based DAVID Bioinformatics Resources database [46]. Gene Set Enrichment Analysis (GSEA) was used for examination of our genome-wide expression profiles [47]. STRING (Search Tool for the Retrieval of Interacting Genes/Proteins) analysis V.11 of functional protein networks was used to define known and predicted protein–protein interactions [48].

### 4.5. DNA Sequencing of the FOXM1 Gene

DNA from parental and resistant cell lines was purified using DNeasy Blood and Tissue Kit (Qiagen, Germantown, MD, USA). Primers were designed to amplify each of the *FOXM1* exons, and amplicons were produced using KAPA HiFi HotStart ReadyMix PCR Kit (KAPA Biosystems, Wilmington, MA, USA). Amplicons were separated on an agarose gel, DNA bands were extracted with the Monarch DNA Gel Extraction Kit (New England Biolabs, Ipswich, MA, USA), and they were subjected to Sanger sequencing.

### 4.6. Our Interferon-Related FOXM1 Inhibitor Resistance Signature (IRFMS), and Clinical Datasets and Kaplan-Meier Survival Analyses

Our Interferon-Related FOXM1 inhibitor resistance Signature (IRFMS) was derived as follows. Eighty percent of the 224 genes in the Molecular signatures Database MsigDB v7.4 Hallmark gene set for Interferon alpha and gamma response were represented in our MCF7 RNA-Seq data, with 152 genes significantly changed, *p* < 0.05. Using a 2-fold gene expression change cut-off, we obtained 43 genes upregulated and 9 downregulated genes. We used the 43 upregulated genes as our IRFMS.

In order to examine the clinical significance of our IRFMS in the prognosis of breast cancer patients, we accessed METABRIC gene expression datasets [49] and analyzed them by using cBioportal (www.cBioportal.org, accessed on 22 October 2021) using Z-score transformed mRNA data and the OncoQuery language function [50,51]. IRFMS signature scores were calculated with mean absolute deviation modified Z-score-normalized mRNA expression data for our gene signature in the METABRIC clinical breast tumor datasets. IRFMS-low, unaltered (−), and IRFMS-high, altered (+), samples were defined by the mean AveMZ score with high being > 2 SD from the mean. Logrank test *p*-values were determined for survival analysis of patients with gene set altered versus gene set unaltered. ER^+^, HER2^−^ clinical samples and TNBC clinical samples were evaluated separately.

### 4.7. Statistical Analyses

Statistics were calculated using analysis of variance (ANOVA), 2-way ANOVA with multiple comparisons, or Student’s *t*-test, as appropriate, using GraphPad Prism 9.0 software. Significance was designated as * for *p* < 0.05, ** for *p* < 0.01, *** for *p* < 0.001, and **** for *p* < 0.0001.

## 5. Conclusions

Due to the fact that FOXM1 is a transcription factor present at high levels in many breast cancers and associated with poor patient outcomes, we examined in this study the effects of FOXM1 inhibition on breast cancer cells and the changes that occur in long-term treated cells that acquire resistance to these inhibitors. Resistant cells upregulate growth factor and inflammatory signaling gene and protein expressions and downregulate ERα and lose responsiveness to antiestrogen, while showing increased sensitivity to lapatinib. A gene signature associated with interferon inflammatory signaling in resistant ER-positive breast cancer cells predicted less good survival in patients with ER-positive breast cancer. Our understanding of how FOXM1 inhibitors suppress breast cancer and how breast cancer cells can undermine their effectiveness and acquire resistance should be helpful in directing further studies to move these agents towards translation into the clinic to benefit patients with breast cancer and possibly other cancers driven by FOXM1.

## Figures and Tables

**Table 1:** Hallmark pathways associated with K-means gene expression clusters A–D in parental, treated, and resistant breast cancer cells.

MCF7				231			
Cluster	Adj. *p*-val.	#Genes	Hallmark Pathways	Cluster	Adj. *p*-val.	#Genes	Hallmark Pathways
A	1.20 × 10^−35^	49	TNFA signaling via NFκB	A	1.05 × 10^−20^	38	TNFA signaling via NFκB
A	5.24 × 10^−25^	27	Cholesterol homeostasis	A	6.68 × 10^−13^	29	Hypoxia
A	1.09 × 10^−24^	39	MTORC1 signaling	A	6.68 × 10^−13^	29	MTORC1 signaling
A	1.41 × 10^−21^	36	Hypoxia	A	1.00 × 10^−11^	21	Unfolded protein response
A	5.29 × 10^−21^	28	Unfolded protein response	A	6.31 × 10^−10^	25	P53 pathway
A	6.97 × 10^−16^	30	P53 pathway	A	1.44 × 10^−8^	23	Interferon gamma response
A	5.44 × 10^−11^	22	Apoptosis	A	5.28 × 10^−8^	22	Heme metabolism
A	2.73 × 10^−9^	16	Androgen response	A	1.97 × 10^−7^	19	Apoptosis
A	1.77 × 10^−8^	21	Estrogen response early	A	4.73 × 10^−6^	13	Interferon alpha response
A	8.68 × 10^−8^	20	Inflammatory response	A	4.73 × 10^−6^	19	IL2 STAT5 signaling
B	1.19 × 10^−59^	72	E2F Targets	B	3.59 × 10^−30^	38	TNFA signaling via NFκB
B	1.50 × 10^−29^	47	G2M Checkpoint	B	2.43 × 10^−15^	25	KRAS signaling up
B	2.79 × 10^−24^	42	Estrogen response early	B	1.30 × 10^−13^	23	Epithelial mesenchymal transition
B	1.40 × 10^−18^	36	Estrogen response late	B	1.30 × 10^−13^	23	Inflammatory response
B	2.53 × 10^−6^	18	UV response up	B	7.84 × 10^−11^	20	Allograft rejection
B	1.51 × 10^−5^	19	Myc targets v1	B	2.15 × 10^−8^	12	IL6 JAK STAT3 signaling
B	1.80 × 10^−5^	16	DNA repair	B	5.97 × 10^−6^	14	Complement
B	4.47 × 10^−5^	18	MTORC1 signaling	B	2.86 × 10^−5^	13	IL2 STAT5 signaling
B	0.000138	17	Glycolysis	B	0.000396	10	UV response up
B	0.00068	8	Myc targets v2	B	0.000539	11	Interferon gamma response
C	9.01 × 10^−12^	19	Interferon alpha response	C	9.55 × 10^−68^	66	G2M checkpoint
C	3.15 × 10^−7^	20	Interferon gamma response	C	3.81 × 10^−66^	65	E2F targets
C	8.73 × 10^−5^	16	Adipogenesis	C	1.36 × 10^−17^	28	Mitotic spindle
C	8.73 × 10^−5^	16	Estrogen response early	C	5.37 × 10^−10^	20	MTORC1 signaling
C	0.000767	14	Epithelial mesenchymal transition	C	5.37 × 10^−10^	20	Myc targets v1
C	0.000767	14	Glycolysis	C	2.02 × 10^−6^	9	Myc targets v2
C	0.000767	14	P53 pathway	C	0.000441	12	Estrogen response late
C	0.006029	10	UV response dn	C	0.000441	12	Glycolysis
C	0.006577	12	Complement	C	0.000601	10	DNA repair
D	0.009467	4	TNFA signaling via NFκB	D	3.10 × 10^−12^	21	UV response dn
D	0.009467	4	KRAS signaling up	D	7.88 × 10^−9^	20	Epithelial mesenchymal transition
				D	3.45 × 10^−8^	19	Estrogen response late
				D	1.60 × 10^−7^	18	Estrogen response early
				D	4.07 × 10^−6^	16	Hypoxia
				D	3.41 × 10^−5^	9	Cholesterol homeostasis
				D	7.08 × 10^−5^	14	Myogenesis
				D	0.00112	12	KRAS signaling up

## Data Availability

All RNA-Seq data have been uploaded to the Gene Expression Omnibus, and they have been assigned GSE accession numbers GSE186679, GSE186682, and GSE186683 that will be publicly available.

## Supplementary Materials

The following are available online at https://www.mdpi.com/article/10.3390/cancers13246282/s1. Table S1: Information on antibodies used in this study; Table S2: IC50 values for inhibition of cell proliferation; Table S3: Statistical analysis of FOXM1 target gene expressions in MCF7 cells treated with NB73 or NB115 for 9 h (goes with Figure 1D) (Excel file); Table S4: Statistical analysis of FOXM1 target gene expressions in MCF7 and in 231 P, T, and R cells (goes with Figure 1E) (Excel file); Table S5: Genes in our IRFMS signature and overlaps with other interferon related resistance signatures; Table S6: Gene-wise statistical analysis for each of the 85 cell cycle related genes in MCF7 and 231 control parental, 24 h treated, and resistant cells (goes with Figure 7). (Excel file); Figure S1: Validation of the expression of a panel of genes from RNA-Seq data and measured by RT-qPCR; Figure S2 Venn diagrams showing overlaps in regulation of gene expressions in several hallmark pathways in response to NB73 in MCF7 and 231 cells; Figure S3: Full-uncut Western blots for Figure 3C, Figure 4B,E, Figure 5C and Figure 6B.
