# Supplementary material for: Transcription Regulation and Genome Rewiring Governing Sensitivity and Resistance to FOXM1 Inhibition in Breast Cancer
Source: Cancers (Basel). 2021 Dec 14;13(24):6282. doi: 10.3390/cancers13246282 (PMC8699539; doi:10.3390/cancers13246282)

Supplementary Materials (Tables and Figures)

Transcription Regulation and Genome Rewiring Governing Sensitivity and Resistance to FOXM1 Inhibition in Breast Cancer

Yvonne Ziegler, Valeria Sanabria Guillen, Sung Hoon Kim, John A. Katzenellenbogen, and Benita S. Katzenellenbogen

Supplementary Table S1. Information on Antibodies used in this study

Name	Company	Product #
ERα	Cell Signaling Technology	8644
FOXM1	Cell Signaling Technology	5436
HER2	Abcam	2428
EGFR	Santa Cruz Biotechnology	sc-03
E2F1	Cell Signaling Technology	3742
CCND1	Cell Signaling Technology	55506
CCNE2	Cell Signaling Technology	4132
CDK4	Cell Signaling Technology	12790
β-actin	Sigma Aldrich	A2228
STAT1	Cell Signaling Technology	14994
p-STAT1-Tyr701	Cell Signaling Technology	9167
STAT3	Cell Signaling Technology	9139
p-STAT3-Tyr705	Cell Signaling Technology	9145

Supplementary Table S2. IC50 Values for Inhibition of Cell Proliferation

Cell, Compound	Expt 1	Expt 2	Expt 3	Mean ± SEM*
MCF-7, NB73	1.20×10^{-6}	0.71×10^{-6}	1.10×10^{-6}	$1.00 \pm 0.15 \times 10^{-6}$
MCF-7, NB115	0.71×10^{-6}	1.20×10^{-6}	0.50×10^{-6}	$0.80 \pm 0.21 \times 10^{-6}$
231, NB73	0.77×10^{-6}	0.43×10^{-6}	0.37×10^{-6}	$0.52 \pm 0.12 \times 10^{-6}$
231, NB115	1.10×10^{-6}	0.48×10^{-6}	0.51×10^{-6}	$0.70 \pm 0.20 \times 10^{-6}$

*No statistical difference between any of these treatments (one-way ANOVA with multiple comparisons). Thus, MCF7 and 231 cells respond similarly to these two compounds, and NB73 and NB115 are similar in potency in suppressing proliferation.

Supplementary Table S3 - Excel file SupplementaryTable_S3_Fig_1D

Supplementary Table S4 – Excel file SupplementaryTable_S4_Fig1E

Supplementary Table S5. Genes in our IRFMS Signature and Overlaps with Other Interferon Related Resistance Signatures

Our 43-gene IRFMS	IRFMS overlap with IRPS-Schiff	IRFMS overlap with IRDS-Weichselbaum	IRFMS overlap with Rad-R and Tam-R-Post	Genes unique to our IRFMS
**IFI44**	**IFI44**	**IFI44**	**IFI44**	CCL2
**IFI27**	**IFI27**	**IFI27**	**IFI27**	TMEM140
**IFIT1**	**IFIT1**	**IFIT1**	**IFIT1**	IFIT2
**IFIT3**	**IFIT3**	**IFIT3**	**IFIT3**	TNFAIP3
OAS2	OASL	OASL	OAS1	SSPN
SAMD9	IFI35	IFI35	OAS3	ICAM1
OAS1	STAT1	STAT1	BST2	PDE4B
CCL2	HERC6	HERC6	OAS2	ISG20
DDX60	IRF7	IRF7	DDX60	TENT5A
TMEM140	SAMD9	OAS1		PARP14
IFIT2	SP110	OAS3		TRIM5
TNFAIP3	IFIH1	BST2		UBE2L6
SSPN	PARP9			NMI
ICAM1	ISG15			ST3GAL5
OAS3	PARP12			HLA-DMA
PDE4B	IRF9			BTG1
OASL	OAS2			CDKN1A
SP110	DDX60			FAS
ISG20				METTL7B
TENT5A				HELZ2
PARP14				TAPBP
IFI35				TAP1
IFIH1				
TRIM5				
PARP9				
ISG15				
UBE2L6				
NMI				
PARP12				
BST2				
ST3GAL5				
HLA-DMA				
STAT1				
HERC6				
BTG1				
IRF7				
CDKN1A				
FAS				
METTL7B				
HELZ2				
TAPBP				
TAP1				
IRF9				

Supplementary Table S6 – Excel file SupplementaryTable_S6_cell_cycle_genes_expression

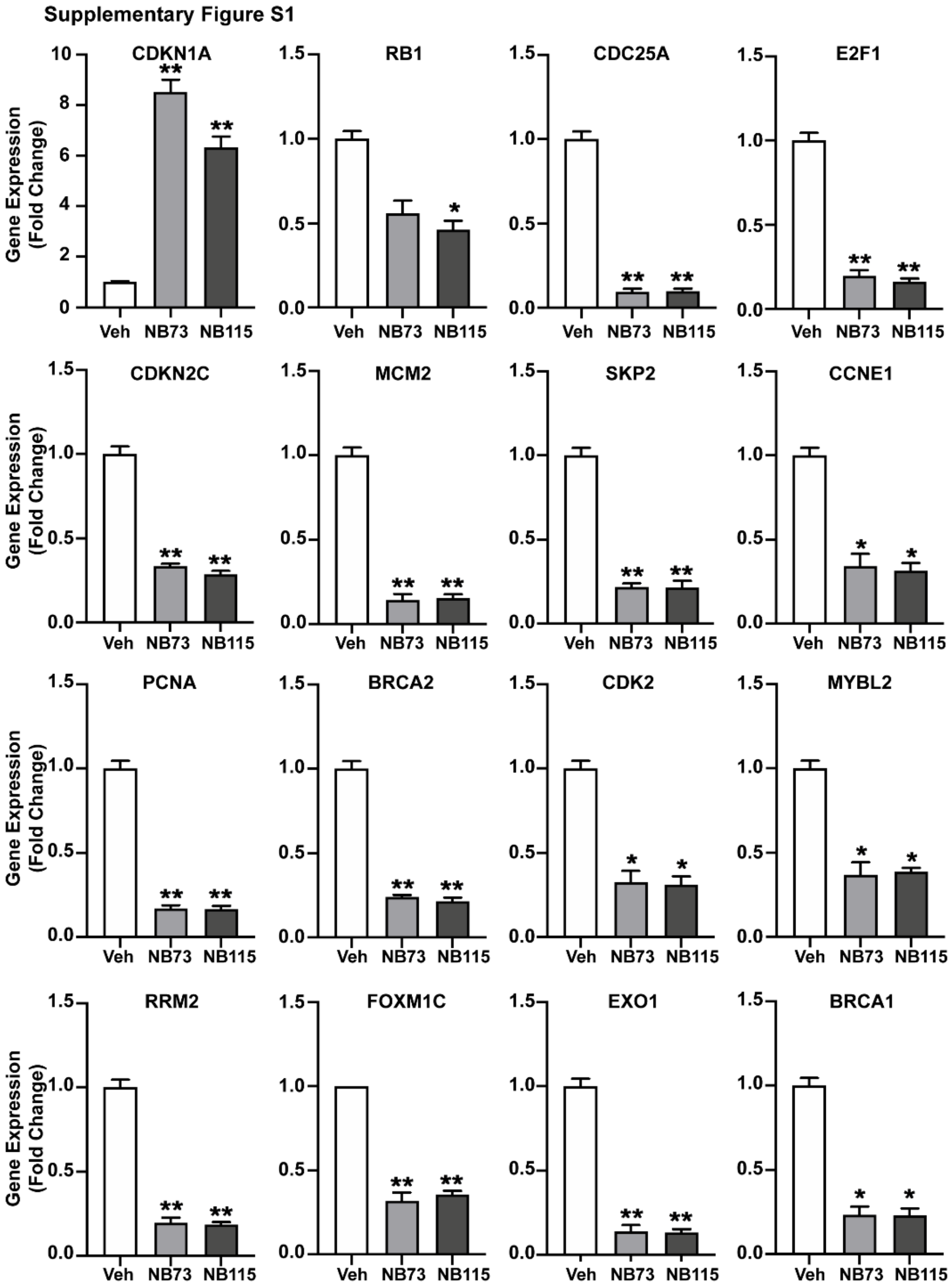

Supplementary Figure S1. Validation of the expression of a panel of genes from RNA-Seq data and measured by RT-qPCR. MCF7 cells were treated with 4μM NB73, 4μM NB115 or Vehicle for 24h. Assays were done in triplicate. Values are mean + SEM.*$p < 0.05$; **$p < 0.01$.

Supplementary Figure S2

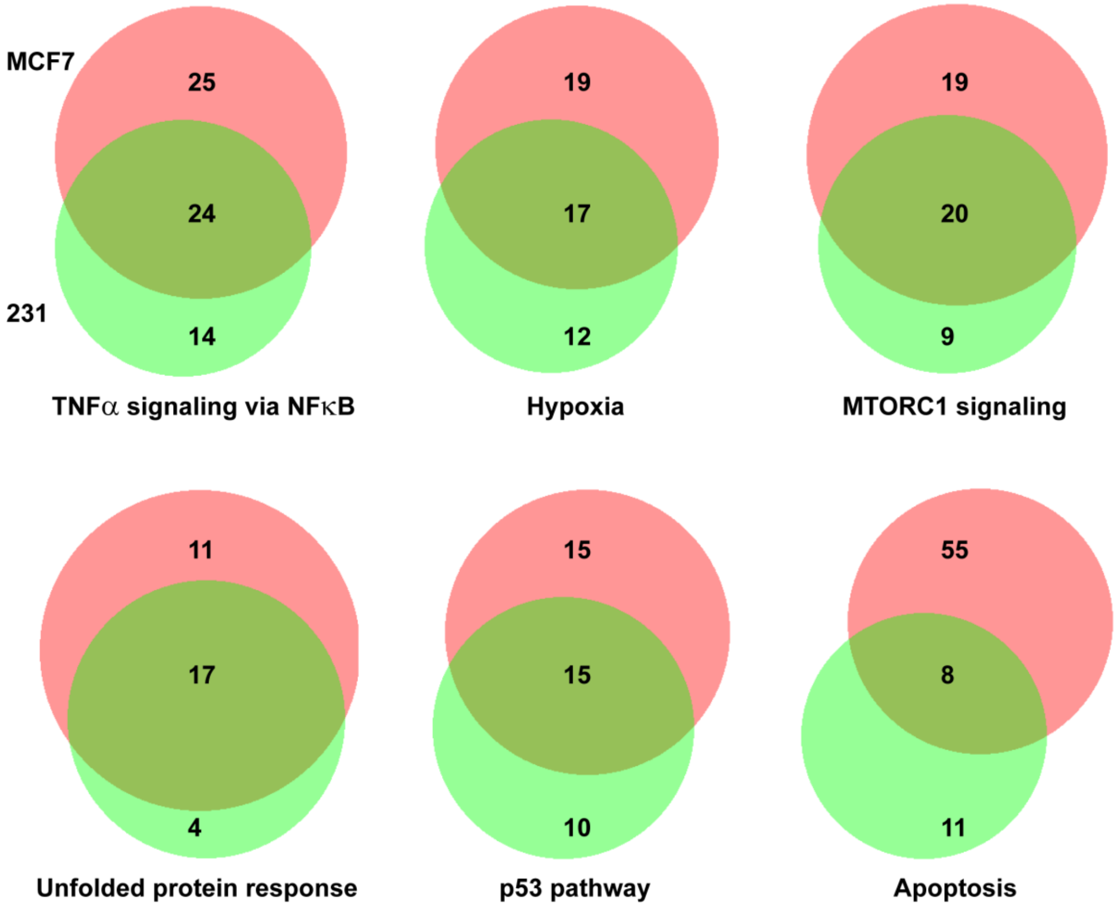

Supplementary Figure S2. Venn diagrams showing overlaps in regulation of gene expressions in several Hallmark Pathways in response to NB73 in MCF7 and 231 cells. This is related to Fig. 2 and Table 1.

Supplementary Figure S3. Full-uncut Western blots for Figures 3C, 4B, 4E, 5C and 6B.

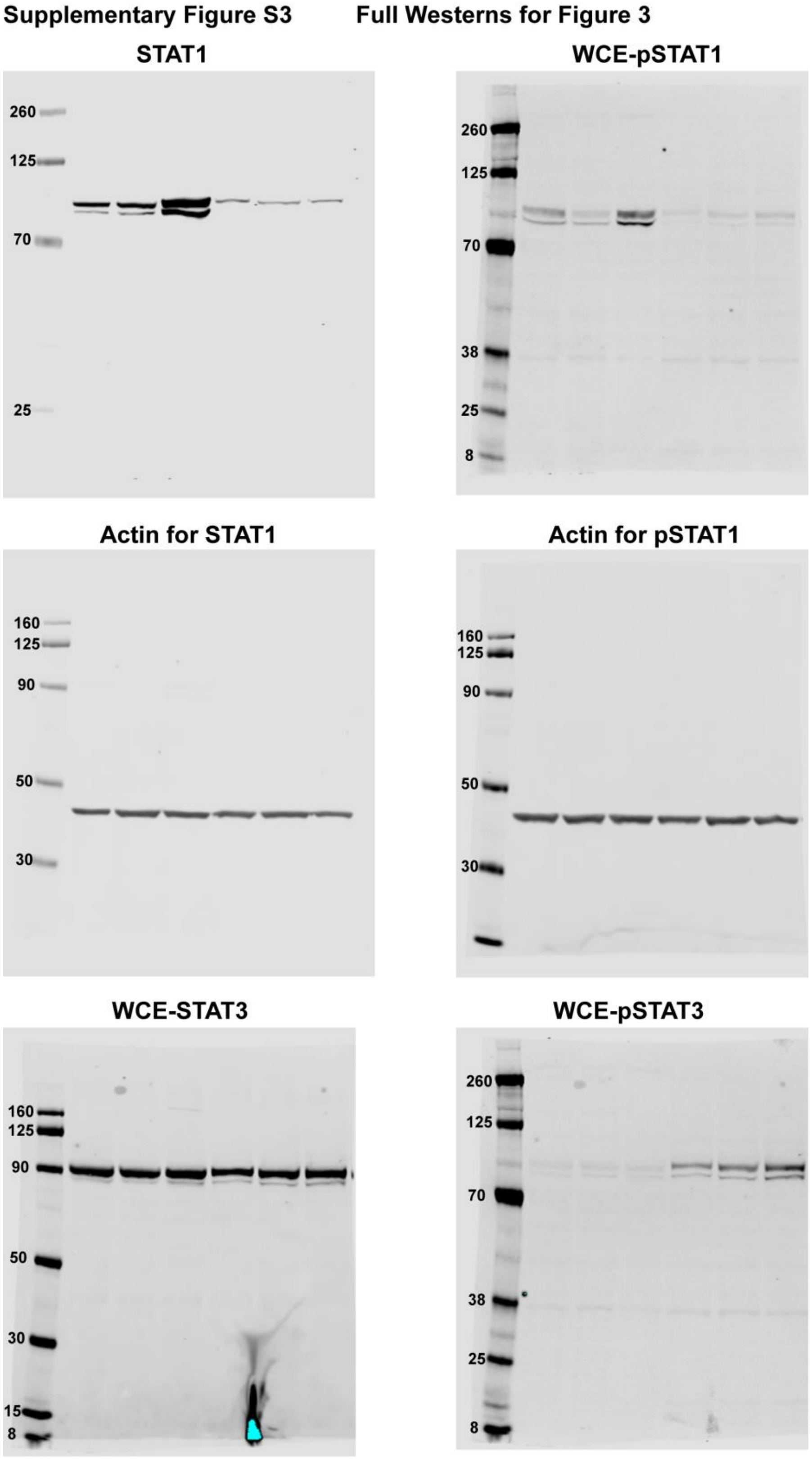

Supplementary Figure S3 **Full Westerns for Figures 4B and 4E**

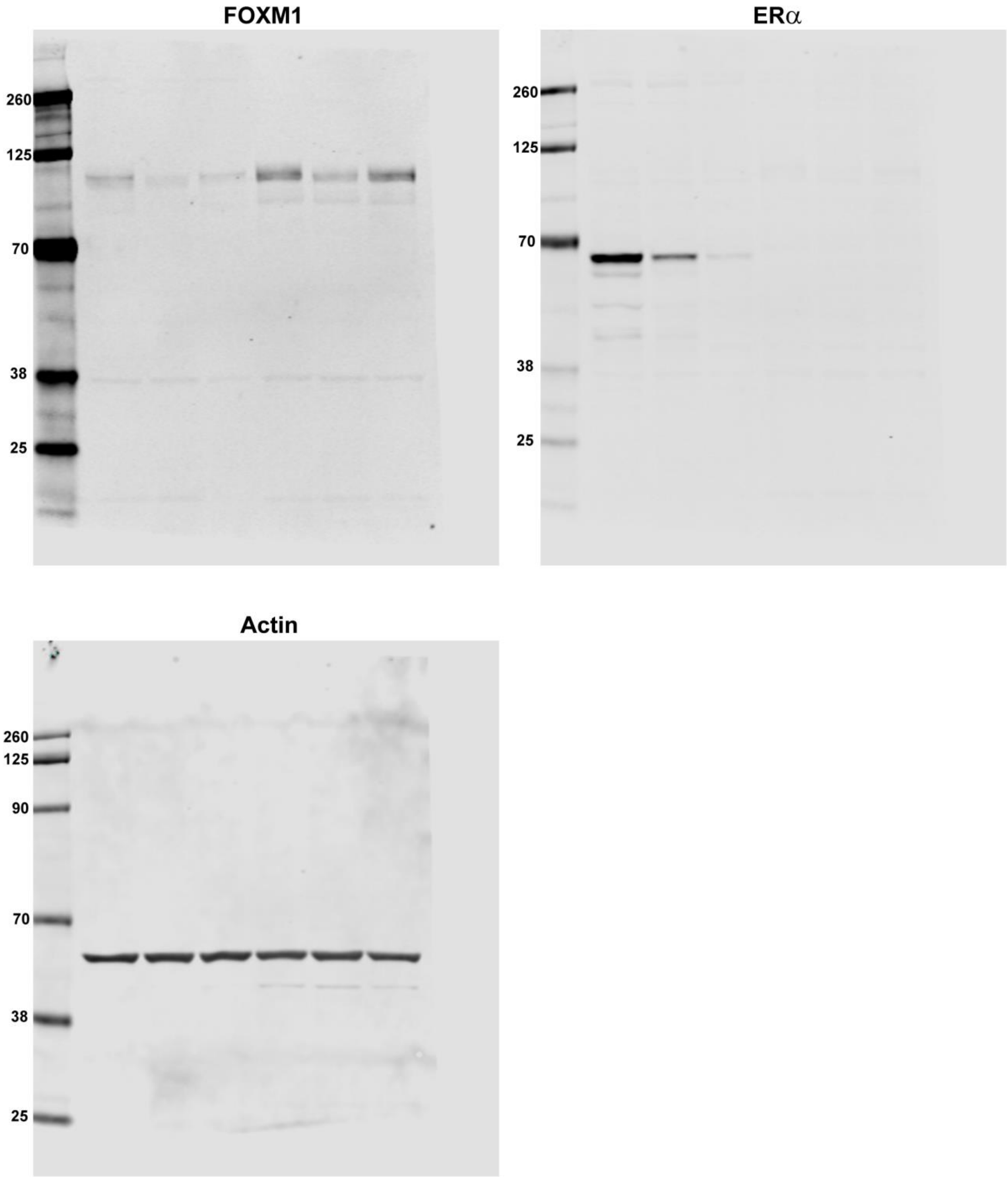

Supplementary Figure S3

Full Westerns for Figure 5

HER2

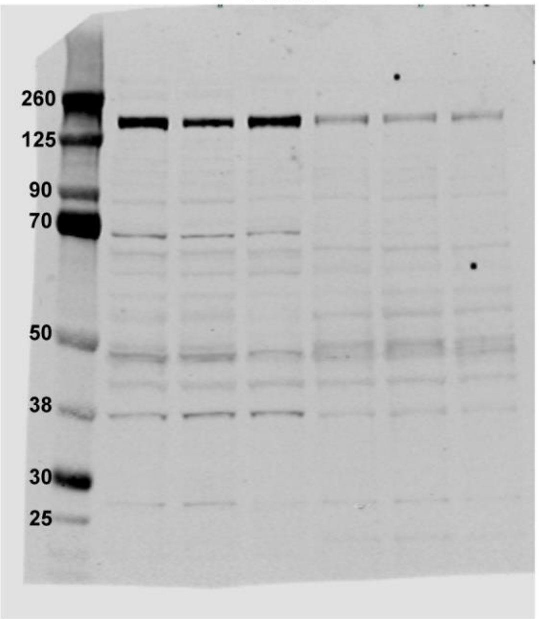

EGFR on stripped STAT3

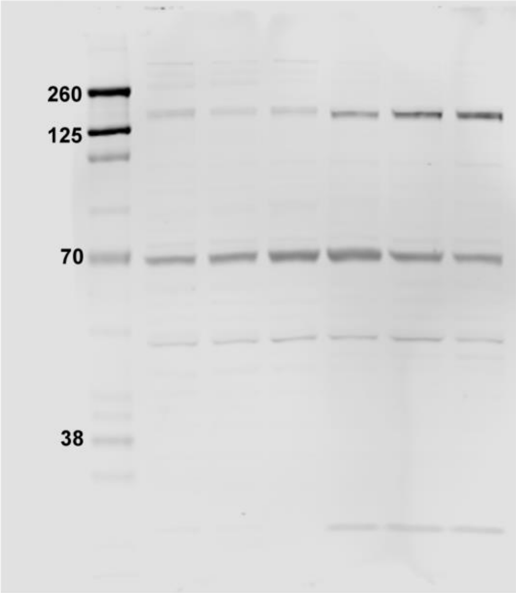

Actin for HER2

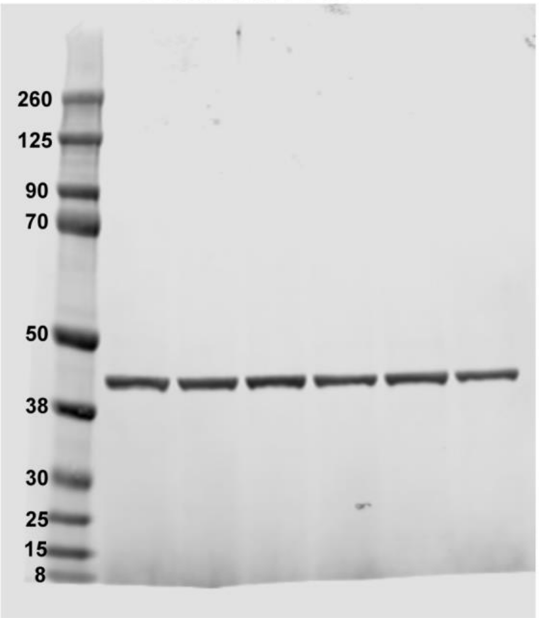

Actin for EGFR

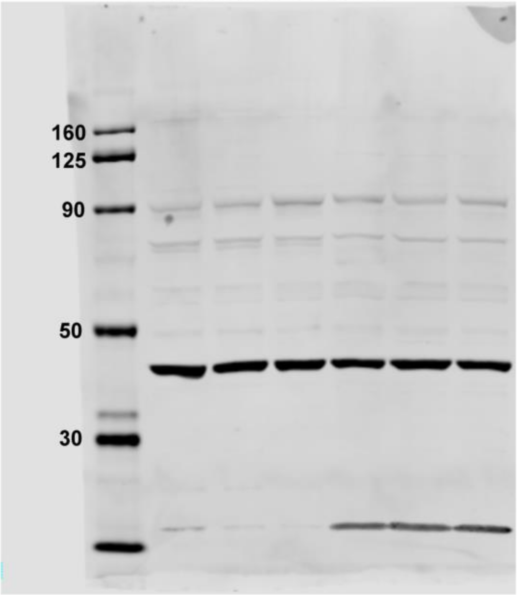

Supplementary Figure S3 **Full Westerns for Figure 6**

E2F1

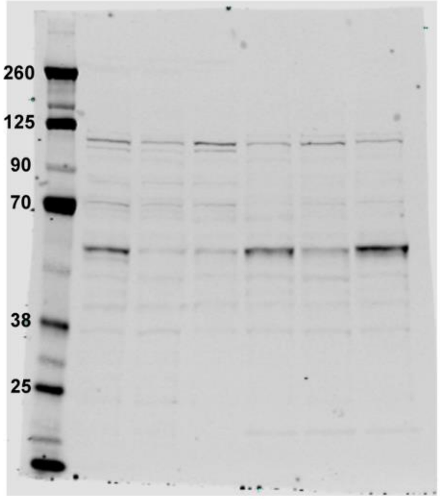

CCND1

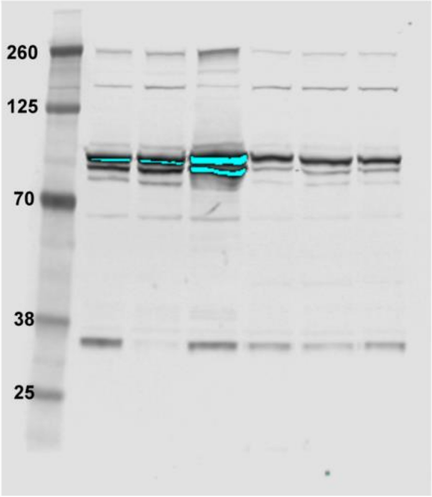

Actin Control

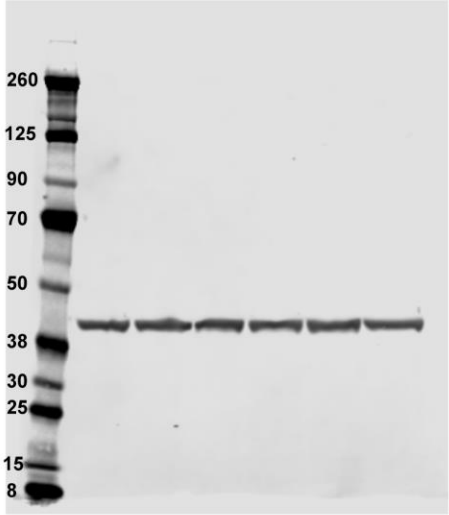

Actin Control

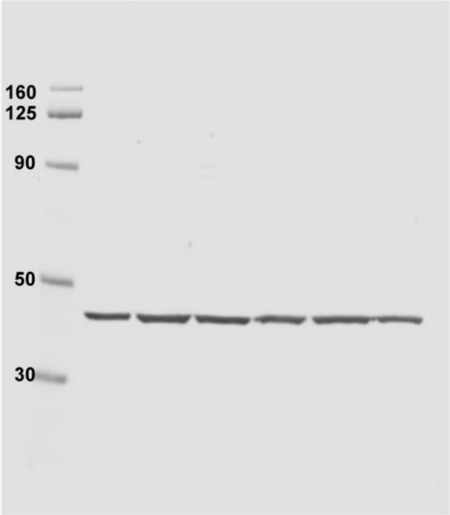

CCNE2, NX

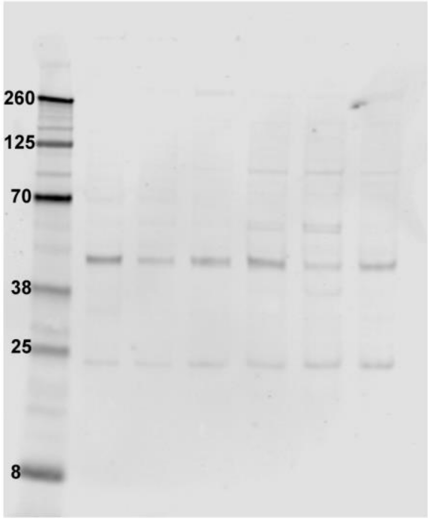

CDK4, NX

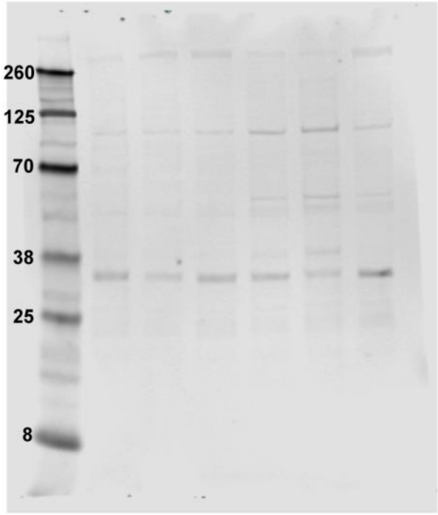

Supplement: Supplementary file 1 [file cancers-13-06282-s001.zip › FOXM1_Supplementary_Materials_120621.pdf]
